# Living conditions and mental wellness in a changing climate and environment: focus on community voices and perceived environmental and adaptation factors in Greenland

**DOI:** 10.1016/j.heliyon.2021.e06862

**Published:** 2021-04-30

**Authors:** Ulla Timlin, Jón Haukur Ingimundarson, Leneisja Jungsberg, Sofia Kauppila, Joan Nymand Larsen, Tanja Nordström, Johanna Scheer, Peter Schweitzer, Arja Rautio

**Affiliations:** a Faculty of Medicine, University of Oulu, Finland; b Stefansson Arctic Institute, Iceland; c University of Akureyri, Iceland; d Copenhagen University, Institute for Geosciences and Natural Resource Management, Denmark; e Nordregio, Stockholm, Sweden; f Department of Civil Engineering, Technical University of Denmark, Denmark; g University of Vienna, Austria; h University of Alaska Fairbanks, USA; i University of Arctic, Finland; j Faculty of Medicine, University of Oulu, Oulu, Finland

**Keywords:** Arctic, Climate change, Indigenous people, Mental wellness, Permafrost thaw, Well-being, Quality of life, Satisfaction with life

## Abstract

**Background:**

Climate change is a major global challenge, especially for Indigenous communities. It can have extensive impacts on peoples’ lives that may occur through the living environment, health and mental well-being, and which are requiring constant adaptation.

**Objectives:**

The overall purpose of this research was to evaluate the impacts of climate change and permafrost thaw on mental wellness in Disko Bay, Greenland. It contained two parts: multidisciplinary fieldwork and a questionnaire survey. The aim of the fieldwork was to learn about life and living conditions and to understand what it is like to live in a community that faces impacts of climate change and permafrost thaw. For the questionnaire the aim was to find out which perceived environmental and adaptation factors relate to very good self-rated well-being, quality of life and satisfaction with life.

**Analysis:**

Fieldwork data was analyzed by following a thematic analysis, and questionnaire data statistically by cross-tabulation. First, the associations between perceived environmental and adaptation factors were studied either by the Pearson χ^2^ test or by Fisher's exact test. Second, binary logistic regression analysis was applied to examine more in depth the associations between perceived environmental/adaptation variables and self-rated very good well-being, satisfaction with life and quality of life. The binary logistic regression analysis was conducted in two phases: as univariate and multivariate analyses.

**Results:**

Nature and different activities in nature were found to be important to local people, and results suggest that they increase mental wellness, specifically well-being and satisfaction with life. Challenges associated with permafrost thaw, such as changes in the physical environment, infrastructure and impacts on culture were recognized in everyday life.

**Conclusions:**

The results offer relevant information for further plans and actions in this field of research and at the policy level. Our study shows the importance of multidisciplinary research which includes the voice of local communities.

## 1 Introduction

The population of the Arctic is estimated to be somewhere between 4 million people (Larsen and Fondahl, 2014) and 7 million (Jungsberg et al., 2019) to 10 million people, depending on how the borders of the Arctic are defined (Emelyanova, 2017). Based on a recent Nordregio Working Paper (Jungsberg et al., 2019) about one million (9%) of the Arctic population identify as Indigenous. In Greenland, most of the population (over 85%) is Indigenous (Ellsworth and O'Keeffe, 2013). Approximately 56.000 people live in Greenland, and the population centers range from small villages with 50–500 inhabitants to the largest town with 17.000 inhabitants in the capital, Nuuk. Settlements are primarily located on the coastline (Bjerregaard and Larsen, 2018).

Traditional knowledge and cultural activities, including traditional occupations of subsistence hunting and foraging, are essential for Indigenous people in the Arctic. These strengthen peoples’ experience of themselves and support contact with nature (Arctic Council Secretariat, 2015; Larsen et al., 2010), as well as material sustenance (Larsen et al., 2010). Adapting to ongoing changes in the environment has always been central for Arctic communities in order to continue their traditional lifestyles (Manrique et al., 2018). Arctic communities are currently experiencing numerous rapid social changes, social changes **-** such as demographic and socio-economic **-** as well as facing a diverse range of challenges due to climate change.

The Arctic is warming at approximately twice the global rate, and especially winter warming events are increasingly frequent and last longer. Humidity and ocean temperatures are increasing, which results in the melting of snow and ice (AMAP, 2019; IPCC, 2014). Ice sheets in Greenland have melted rapidly during the years 2002–2011, but also the decrease of glaciers, ice-sheets and snow cover have been fast (IPCC, 2014). In addition, permafrost thaw and coastal erosion are damaging buildings and infrastructure (AMAP, 2019; Bronen and Chapin, 2013) and putting historical heritage at risk (Hollesen et al., 2018). The thawing of permafrost is also causing health risks for people (AMAP, 2019; Brubaker et al., 2011; Watts et al., 2015). Changes in the surrounding environment are causing problems for physical and mental health, and people may suffer from various mental symptoms (Dodgen et al., 2016), such as depression, anxiety, grief and various stress disorders (Bourque and Cunsolo Willox, 2014; McMichael, 2013).

Impacts on human health and well-being can be divided into direct and indirect impacts. Direct impacts are often related to acute and sudden changes in climate, such as storms, floods or fires in nature, resulting in physical injuries, even deaths and symptoms of stress (Bourque and Cunsolo Willox, 2014; McMichael, 2013; Watts et al., 2015). Further, climate-related impacts on human health may occur in several ways, such as the breakout of (new or old) infections or diseases (Waits et al., 2018; Watts et al., 2015), usually through contaminated food and water (Brubaker et al., 2011; Watts et al., 2015; Cunsolo Willox et al., 2012). Many climate-sensitive diseases are zoonotic, i.e. caused by viruses, bacteria or parasites that spread between animals and people (Waits et al., 2018). Additionally, injuries and serious mental burden among people may be increased (Brubaker et al., 2011; Watts et al., 2015), and financial impacts can occur (Bronen and Chapin, 2013; Dodgen et al., 2016). These can be connected to loss of livelihoods and damages to the living environment, as well as infrastructure (Bourque and Cunsolo Willox, 2014; McMichael, 2013).

Indigenous communities in the Arctic can be in an especially vulnerable situation with the multiple changes in the climate, in their living environment and their way of life (Ford et al., 2014; Nuttall, 2010). They are connected holistically to their home, environment, nature, and a variety of commitments (Chatwood et al., 2017). For Inuit, well-being is holistic, including enjoying a supportive family life, talking, and having intimate communication and adhering to traditional culture, ways of life and connection to the land (Kral and Idlout, 2012). For Indigenous people living in Greenland, hunting and fishing are an essential part of a traditional way of life (Pedersen, 2007). According to Drivsholm (2017) one element supporting the culture in Greenland is the use of sled dogs. It has been a natural part of culture providing for the possibility to travel, hunt and fish. However, this culture is being challenged due to climate change as the sea ice and snow cover are decreasing, and the use of snow mobiles instead of sled dogs is becoming more common (Drivsholm, 2017). This trend may have further impacts on the use of the environment in Greenland and on the health and well-being of people (Sonne et al., 2018).

Much of the research investigating climate change impacts on the health and well-being of Indigenous people have mainly focused on people living in North America and Northern Canada, e.g. studies completed by Brubaker et al. (2011), Ford et al. (2014), Ford et al. (2010), Cunsolo Willox et al. (2012) and Cunsolo Willox et al. (2013b). More research is needed on the impacts of permafrost thaw on peoples’ lives and mental well-being in Greenland. Our research contained two phases. The first phase involved multidisciplinary fieldwork in February 2019 to build awareness and understanding of the life and living conditions from the perspectives of local communities. The second phase involved a multidisciplinary questionnaire, collected in the spring of 2019, to collect survey data regarding self-rated well-being, quality of life, and satisfaction with life under climate change and thawing permafrost in the Disko Bay area. These constructs form elements of mental wellness. The overall purpose of our research was to evaluate the impacts of climate change and permafrost thaw on mental wellness.

**Figure 1:**
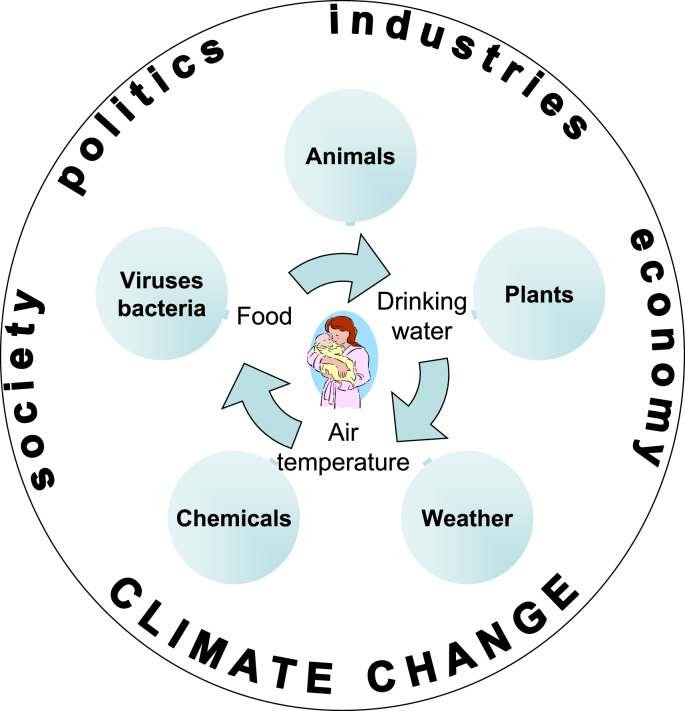
**Descriptionof** the elements of well-being by Alasaarela and Rautio in the publication Rautio (2009).

Our study of the impacts of permafrost thaw located in Disko Bay, Greenland, is part of the multidisciplinary EU Horizon 2020 funded project named “Nunataryuk”. The “One Health” approach was used as a framework as it focuses holistically on the health of humans, environment, animals, and plants (Ruscio et al., 2015) (See Figure 1).

## 2 Material and methods

### 2.1 Data collection

#### 2.1.1 Fieldwork

The fieldwork in Disko Bay was carried out over a period of two and a half weeks in two communities by a team of researchers from the academic disciplines of health sciences, social sciences and civil engineering. The specific aim of the fieldwork was to learn about life and living conditions and to understand what it is like to live in community that faces impacts of climate change and permafrost thaw. In addition, the fieldwork provided a solid background for future co-operation between local people and researchers.

The data from individual and group discussions were gathered ahead of or during the fieldwork period from community consultation meetings and workshops composed of invited local people who represent their community, utilizing snowball sampling. In the period before the fieldwork, the researchers contacted the leader of the Arctic Station who helped organize and host a formal community-consultation meeting. He also assisted with both soliciting participants that broadly represented the community as well as hiring a local research assistant who served as an interpreter and became a key informant for the research team. Participants for the consultation meeting were gathered through local contacts with the research assistant, and also via recommendations received from those who had participated in the initial workshop co-organized with the municipality. In total, researchers had discussions with approximately 60 adults, of which more intensive discussions were conducted with 53 people. The level of discussions varied as discussions were often more informal, with the number of participants changing while meetings were ongoing. One round of discussions was conducted.

The researchers also visited a local elementary school and introduced informally the project to a small group of the teachers and the principal. They subsequently engaged these teachers and the principal in discussions concerning lifestyles and living conditions within the community, with a primary focus on issues of particular relevance for children and young people. The researchers were also invited to conduct a 1-h class with about 30–40 schoolchildren (ages 12–15 years), where the project was presented and various themes were discussed, emphasizing key issues in the lives of the school children, and their views and perspectives on climate change in the region. Some of the questions posed, and issues raised were based on the aims of the project, while others were based on the Arctic Youth and Sustainable Futures project (Stefansson Arctic Institute, 2019). This was a good opportunity for researchers to inform children about climate change, but also for researchers to learn from the children. No specific data were collected from the children, but the session in the classroom helped researchers to learn more about life and living conditions in the community.

The data gathered during this fieldwork did not include any personal health information, just an overall description of experiences of life and living conditions. A consent form to be signed prior to discussions was developed ahead of the fieldwork with all names and contact information of the research team included. The consent form was used in individual and group discussions. It was signed by a study representative and a copy of the consent form was offered to the community representative. Information about the project was presented by the researchers and summary information was provided in the written consent form. In the elementary school, one of the schoolteachers signed the form on behalf of the school. In the group meetings and workshops, permission was provided by community representatives verbally. They also gave permission for taping the group discussions.

Researchers worked jointly as a multidisciplinary team and in a manner that showed full respect for the local community and they gathered much of discussion data as a team without causing any additional burden to the local people. This process started before the fieldwork and continued during it. Researchers processed relevant themes and questions, and discussions with the communities were carefully planned in advance to help limit overlaps, repetitions of questions, and requests for interviews by different researchers. An interpreter (Danish speaking) was used in the meetings where local people spoke Greenlandic, and researchers who spoke Danish translated the content to other researchers in English. Recordings were transcribed by team members and checked for accuracy of content by the team prior to being utilized. No raw data and no information outside the project's research questions were shared. Only members of the research group had access to the data based on notes, and access was protected by passwords.

Our research plan did not require ethical approval on human or health sciences according to the Finnish National Board on Research Integrity TENK, 2019 (https://www.tenk.fi/en/ethical-review-in-human-sciences). However, the research group has an ethical approval from the Health Research Ethical Committee in Greenland for further research.

#### 2.1.2 Questionnaire

The questionnaire was developed to fulfill the specific need of the multi-disciplinary project. Several references were utilized when creating the questions (Nordic Council of Ministers, 2015; Nordic Council of Ministers, Nordic Council of Ministers Secretariat 2015a; Nordic Council of Ministers, Nordic Council of Ministers Secretariat 2015b). The questions were chosen and finalized based on the knowledge collected from previous fieldwork related to permafrost thaw and climate change. The questionnaire was translated from English to Greenlandic, and pre-tested. After the translation the research assistant made three comments to improve the material and enhance local understanding. Specific changes were made as follows: Firstly, on the order of question one and two, and secondly changes were made to questions that are not included in this present research (extra options for the answers were added on two questions). Local knowledge was utilized to ensure relevance to cultural and language sensitivity. More specific principles for the translation process can be found from the guidelines by World Health Organization (https://www.who.int/substance_abuse/research_tools/translation/en/). Although the same questionnaire was used in other case areas of Nunataryuk, it was essential that necessary small changes could be made to suit the context of each case area. The questionnaire included three topics: 1) changes related to permafrost thaw in the surrounding environment that impact local peoples’ lives; 2) impacts of permafrost thaw on hunting, fishing and harvesting; and 3) background information about participants to obtain relevant demographic variables.

Questionnaire data were gathered in one community (around 1000 residents, including around 200 under the age of 18) located in Disko Bay, which lies in a zone of continuous and discontinuous permafrost (Junsberg et al., 2019). A local research assistant collected the data from February to April 2019, in collaboration with the Arctic Station of the University of Copenhagen. The answers were collected using a combination of snowball sampling and the local research assistant's knowledge about the people living in the community. We encouraged an equal gender balance and representation from all age groups over 18 years. Data were collected face-to-face, using a printed questionnaire that participants were able to fill out themselves. Participation was anonymous, voluntary, and participants were able to refuse participation, skip questions or terminate the process if they wished. The focus of the questionnaire was to investigate self-rated well-being, quality of life and satisfaction with life; and the specific aim was to find out which perceived environmental and adaptation factors relate to very good self-rated well-being, quality of life and satisfaction with life.

### 2.2 Data analyses

#### 2.2.1 Fieldwork data

The data gathered during community meetings and discussions were analyzed by following thematic analysis. The analysis began by reading the entire data set several times to become familiar with the data and to understand the content. This was followed by the identification of all relevant data that offered answers to the research questions. The data were moved into a new file, followed by reading it over several times and then continuing with listing and summarizing the relevant information in order to form an impression of the emerging themes. Finally, the data were reviewed, and emerging themes were identified and named. A more specific description of the thematic analysis can be found from Braun and Clarke (2006). The joint data were analyzed with the following questions in mind: “what it is like to live in this community?” and “what elements describe living conditions, with possible impacts of climate change and permafrost thaw?” As the discussion data consisted of fieldwork notes, written by researchers, no authentic transcriptions are presented in the results.

#### 2.2.2 Questionnaire

Variables describing elements of living conditions and well-being have been used before in large surveys, such as the Survey of Living Conditions in the Arctic (SLiCA, 2015) where variables were used in a larger setting with specific aspects to describe living conditions and well-being (Nordic Council of Ministers, 2015; https://iseralaska.org/static/living_conditions/). Therefore, based on SLiCA, variables of well-being, quality of life and satisfaction with life were chosen for our questionnaire as independent variables to receive information about local peoples’ assessment of what constitutes elements of mental wellness.

Health and well-being can be seen as a combination of mental, physical, spiritual and social well-being (Rautio et al., 2014). The World Health Organization (WHO, 2020) defines mental health as “*a state of well-being in which a person is able to realize his or her own potential, is able to cope in life with normal stress, and is able to work and contribute to his or her community. It is not just an absence of disease or infirmity”* (WHO 2014). Additionally, Ryan and Deci (2001) present mental well-being through two perspectives: 1) feelings of happiness and life satisfaction, and 2) good social functioning and self-esteem. It is essential to recognize how health and well-being and climate change are connected to each other and understand the context in which people are facing changes and adapting to them (Fresque-Baxter and Armitage, 2012). Based on these definitions, it was important that participants were able to self-rate their well-being, quality of life, and satisfaction with life using the Likert scale (very bad, bad, OK, good, very good).

Questions that were chosen to describe and explain connections and impacts of permafrost thaw on mental wellness reflected the following perspectives: 1) use of the natural environment for different activities (hunting, fishing, picking berries and mushrooms, economic, recreation and scientific activities), 2) problems and impacts that thawing is causing, 3) challenges associated with different aspects of life (hunting and harvesting, physical environment, human health, culture, economic activities and housing, and infrastructure, such as buildings and roads), 4) need of adaptation to permafrost thawing by individuals, communities, and local, regional, national and global authorities, and 5) impacts on obtaining food and daily supplies, and 6) demographic variables. These questions included valuable information about the impacts related to the environment and adaptation of local people, which are commonly described in the literature. Specific questions are presented in Appendix 1.

### 2.3 Statistical analysis

Very good well-being, satisfaction with life and quality of life, as compared to not very good (bad, OK, good) were used as dependent variables in the statistical analyses. This context-based classification was used since only a small part (3–14 %) of 100 participants rated their mental wellness as bad or OK. Since the second largest group of participants answered, “very good well-being, quality of life and satisfaction with life”, the variables were dichotomized as very good vs. not very good. This decision was supported due to the small number of participants, but it was also supported by the impression researchers got from the fieldwork (Table 1). There was only one participant who did not answer these questions of well-being, quality of life and satisfaction with life. This one missing value for each dependent variable was replaced with a mean value of good level 4 (well-being: 4.15, quality of life: 4.14, satisfaction with life: 4.36).

**Table 1:** Demographic characteristics/variables and self-informed variables describing mental wellness (n = 100).

Demographic variables	n (%)
**Age**	
18-24	10 (10)
25-34	20 (20)
35-44	11 (11)
45-54	28 (28)
55-64	22 (22)
≥65	9 (9)
**Main profession**	
Public sector	38 (38)
Private sector	31 (31)
Hunter/fisherman∗	11 (11)
Not employed	20 (20)
**Language at home**	
Greenlandic	90 (90)
Danish	5 (5)
Both	5 (5)
**Variables of mental wellness**	
**Self-rated well-being**	
Very good	28 (28)
Good	58 (58)
Bad/OK	14 (14)
**Self-rated quality of life**	
Very good	21 (21)
Good	71 (71)
Bad/OK	8 (8)
**Self-rated satisfaction with life**	
Very good	38 (38)
Good	59 (59)
Bad/OK	3 (3)

∗ Only males in this data were hunters and fishermen.

Relationships between independent variables were analyzed by cross-tabulation, and the associations were tested either by the Pearson χ^2^ test or by Fisher's exact test. Furthermore, binary logistic regression analysis, using the enter method, was used to find possible associations between perceived environmental/adaptation variables and very good well-being, satisfaction with life and quality of life. Variables having an association with either very good well-being, satisfaction with life or quality of life with a *p*-value ≤ 0.1 were included in the models (Table 2).

**Table 2:** Associations of variables describing mental wellness and demographic/perceived environmental factors (*p* = ≤0.06).

	Wellbeing		*p*-value
	not very good	very good	
	n (%)	n (%)	
**Age** (n = 100)			
18-24	8 (11)	2 (7)	**0.031**
25-34	9 (12,5)	11 (39)	
35-44	9 (12,5)	2 (7)	
45-54	20 (28)	8 (29)	
55-64	20 (28)	2 (7)	
≥65	6 (8)	3 (11)	
**Being in nature for picking berries, mushrooms** (n = 99)			
never, rarely, sometimes	31 (44)	19 (68)	**0.012**
very often	40 (56)	8 (29)	
N/A	0 (0)	1 (3)	
**Enough done to adapt/face the impacts, by local authorities** (n = 85)			
no	50 (84)	17 (68)	0.057
somewhat	5 (8)	7 (28)	
yes	5 (8)	1 (4)	
**Challenges are associated with health** (n = 99)			
not important, little important	4 (6)	7 (25)	**0.021**
important, very important	29 (41)	8 (29)	
N/A	38 (53)	1b3 (46)	
	**Quality of life**		p-value
	not very good	very good	
	n (%)	n (%)	
**Language at home** (n = 100)			
Greenlandic	75 (95)	15 (71)	**0.001**
Danish	0 (0)	5 (24)	
Both	4 (5)	1 (5)	
**Main profession** (n = 100)			
Public sector	27 (34)	11 (52)	**0.030**
Private sector	22 (28)	9 (43)	
Hunter/fisherman	10 (12)	1 (5)	
Not employed	20 (26)	0 (0)	
	**Satisfaction with life**		p-value
	not very good	very good	
	n (%)	n (%)	
**Language at home** (n = 100)			
Greenlandic	60 (97)	30 (79)	**0.004**
Danish	0 (0)	5 (13)	
Both	2 (3)	3 (8)	
**Main profession** (n = 100)			
Public sector	22 (35)	16 (42)	**0.004**
Private sector	14 (23)	17 (45)	
Hunter/fisherman	7 (11)	4 (11)	
Not employed	19 (31)	1 (2)	
**Being in nature for recreation activities** (n = 100)			
never, rarely, sometimes	29 (47)	10 (26)	0.057
very often	33 (53)	28 (74)	
**Being in nature for scientific activities** (n = 97)			
never, rarely	56 (95)	33 (87)	**0.027**
sometimes, very often	1 (2)	5 (13)	
N/A	2 (3)	0 (0)	
**Challenges are associated with culture** (n = 99)			
not important, little important, important	26 (43)	10 (26)	**0.005**
very important	8 (13)	16 (42)	
N/A	27 (44)	12 (32)	
**Challenges are associated with housing, buildings, roads** (n = 97)			
not important, little important, important	25 (42)	17 (46)	**0.002**
very important	6 (10)	13 (35)	
N/A	29 (48)	7 (19)	
**Challenges are associated with physical environment** (n = 99)			
not important, little important, important	28 (46)	13 (34)	**0.049**
very important	8 (13)	13 (34)	
N/A	25 (41)	12 (32)	

*p*-values ≤ 0.05 are written in bold.

The analysis was conducted in two phases, firstly univariate analysis and secondly, multivariate analysis adjusted for demographic variables (age, gender, language, and employment situation). On prior logistic regression analysis, the Spearman's rank correlation coefficient values were checked to find correlations of independent variables. In addition to the variables presented in Table 2, variables classified as “Thawing is causing problems” and “Lost picking spots” were included in the model as well. All variables associated with dependent variables (*p*-value ≤ 0.1) are presented in the Appendix tables. The variable for main professional activity was reclassified as follows: public (e.g. teachers and other professionals working in the public sector), private (e.g. private business owner or working as a salesman), hunter or fisherman, and not currently working (e.g. student, unemployed, or retired). The data has been analyzed using IBM SPSS Software, version 25. All tests were two-tailed and *p*-values less than 0.05 were considered statistically significant.

Additionally, all dependent variables were grouped into three classes: bad, OK/good/very good. Results of this classification are presented in the supplemental tables (see Appendix Tables 1–3) and are similar to the reported results here.

## 3 Results

### 3.1 Fieldwork

Based on the fieldwork data, the following interrelated themes were found: 1) Good everyday life, 2) Living challenges, 3) Changing nature and environment, 4) Uncertain future, and 5) Bright future.

*Good everyday life* includes traditional life and living habits of Greenlandic Indigenous people, following their traditional lifeways, culture, and roles. People enjoyed Greenlandic food and considered it healthy, and the occupations of fisherman and hunter were rated highly in society. The traditional hunter and gatherer society appeared to be strong, supporting the Indigenous lifestyle; women worked together doing their own traditional and cultural activities while the men worked together in their sphere. The home community was felt to be calm and a good place to live, and people felt living there was safe overall. Family relationships appeared to be strong, supporting well-being. Additionally, nature was very important and powerful for residents. Wintertime weather was challenging, but still part of everyday life. Overall, residents were not worried about climate change and felt that ordinary people did not talk about it, stating only outsiders like researchers were discussing it. They have had to adapt to changes in the environment and nature. Many respondents felt that there is no problem with permafrost thaw.

*Living challenges* are perceived difficulties in life*.* Residents felt at times isolated, and this feeling strengthened during the wintertime due to difficulties in travelling. Young people could suffer from loneliness, and they did not have many career or educational options to choose from locally. Also, young people in the community could be more isolated nowadays, and one possible reason brought up by adults was the current active use of social media or computer games. Adults also expressed their concerns that many traditional cultural values that used to be culturally important are under threat or had been lost among the youth. Additionally, some had a feeling that families are generally getting smaller. People suffered from gossiping, which made it challenging to express their own problems or concerns to others, even to professionals. Residents wished for greater availability of health and social care professionals with an ability to speak Greenlandic in order to support everyone, including people living in rural areas and those suffering from specific problems. Residents felt they had little leverage on decision-making in the municipality, and that a small group had too much power over decision-making. Economic differences between people were felt to be growing. People were distressed by the lack of jobs, especially during wintertime, which coincides with the low tourist season. Residents were concerned with the poor quality of the infrastructure, which can partly be related to construction practices and lack of financial resources.

*Changing nature and environment* was present in discussions with local residents. They observed a thinning and reduction of the sea ice during wintertime, a reduced snow cover duration, and enhanced loss of land-based ice in summertime. These observations caused worries about the continuation of the traditional Greenlandic culture and it made the traditional use of dog sleds much more difficult and dangerous. A change in wind was also noticed, noting it was more humid and felt stronger. Additionally, risks of natural disasters such as avalanches and tsunamis were noticed. New administrations and policies caused uncertainty and stress, especially among people with traditional occupations, which often provided the only source of income. Even if permafrost thaw did not appear to be a problem for some, this could change depending on living location as there were still worries about the infrastructure damage due to harsh meteorological conditions. This may also be linked to the temporary nature of the repairs. Finally, the lack of data regarding permafrost conditions was seen as a problem as well.

*Uncertain future* was reflected in the lack of capacity, infrastructure and preparations for the future. Residents could have a feeling of sadness and anxiousness due to all the changing conditions in their lives. Growing tourism could create new challenges and demands on the community and environment, including search and rescue preparations for accidents and responses to natural disasters. Additionally, they had worries about the loss of a peaceful environment, but also growing tourism that caused concerns about the competition for labor. Generally, the lack of jobs in the future worried residents. They expressed a desire for more highly educated people, knowledge, resources and effective cooperation between professionals. Residents worried that bigger places are growing while rural communities are becoming smaller, creating inequality between people living in the same municipality. More detailed site-specific information and decision support tools would benefit planning and building strategies.

The notion of a *bright future* seemed to support the life of local people. People felt they had a high quality of life and were generally healthier nowadays, due to the wealth of information that was currently available. Access to the Internet was felt to be helpful. Generally, residents felt they needed to adapt to new situations and challenges. All changes could also create new possibilities and opportunities, and the changing climate could help tourism to grow. People saw the growing tourism business as a good thing as it created new possibilities both locally and financially.

### 3.2 Questionnaire

Altogether 100 participants (48 women, 52 men) took part in the questionnaire survey. Most of the participants were aged 45–54 years (Table 1). Independent variables that have a statistically significant association with dependent variables are shown in Table 2. Main professional activity was significantly associated with self-rated satisfaction with life (*p =* 0.004) and quality of life (*p* = 0.030). A significant association was also found between well-being and age (*p* = 0.031). Participants aged 24–35 formed the largest group of participants with a very good well-being (n = 11, 39 %). The majority that replied with not a very good well-being were aged 45–54 years (n = 20, 28 %) and 55–64 years (n = 20, 28 %).

#### 3.2.1 General description of self-rated well-being, quality of life and satisfaction with life

Recreational activities in the natural environment seemed to support satisfaction with life (*p* = 0.057). Participants who self-rated a very good satisfaction with life were in nature for recreational activities very often (n = 28, 74 %) compared to those who were less often (n = 10, 26 %). However, picking berries or mushrooms did not support well-being. The majority of participants with very good well-being (n = 19, 68 %) did not pick berries or mushrooms (*p* = 0.012) compared to participants with a not very good well-being (n = 40, 56 %), who picked berries and mushrooms very often. Challenges associated with health were recognized as being more common among participants with poorer well-being. Participants with a very good well-being assessed challenges associated with health as important or very important (n = 8, 29 %) compared to participants with not a very good well-being (n = 29, 41 %). Even when a statistically significant association was found (*p* = 0.021), a large group of participants answered N/A to this question (not very good: n = 38, 53 %, very good: n = 13, 46 %). Challenges associated with culture were seen as very important among participants with a very good satisfaction with life (n = 16, 42 %, *p* = 0.005). The majority of participants among the group with not very good well-being (n = 50, 84 %) felt not enough has been done by local authorities to adapt to or face the impacts of permafrost thaw compared to those who rated their well-being as very good (n = 17, 68 %). This finding was not statistically significant (*p* = 0.057) (See Table 2.).

#### 3.2.2 Variables associated with very good well-being, quality of life and satisfaction with life

Table 3 shows the results of a univariate binary logistic regression analysis examining the association of perceived environmental and adaptation factors with very good well-being, quality of life and satisfaction with life. Perceived adaptation factors were not significantly related to any of the dependent variables. In addition, the results of the multivariate regression analysis examining the association of perceived environmental factors with dependent variables are presented in the Supplement table 4. The results are similar to the presented results of the univariate analysis.

**Table 3:** Univariate analysis: Associations between variables describing mental wellness and perceived environmental variables (*p* = <0.06).

Variables	yes (n/%)/total	OR	95 % CI	*p-*value
	Very good well-being			
**Being in nature for picking berries, mushrooms**				
never, rarely, sometimes	19 (38 %)/50	3.07	1.19–7.92	**0.021**
very often	8 (17 %)/48	Ref.		
**Challenges associated with health**				
not important, little important	7 (64 %)/11	6.34	1.48–27.22	**0.013**
important, very important	8 (22 %)/37	Ref.		
	**Very good quality of life**			
**Challenges associated with culture**				
not important, little important, important	4 (11 %)/36	0.25	0.07–1.0	**0.043**
very important	8 (33 %)/24	Ref.		
	**Very good satisfaction with life**			
**Being in nature for scientific activities**				
never, rarely	33 (37 %)/89	0.12	0.01–1.05	0.056
sometimes, very often	5 (83 %)/6	Ref.		
**Being in nature for recreation activities**				
never, rarely, sometimes	10 (26 %)/39	0.41	0.17–0.98	**0.044**
very often	28 (46 %)/61	Ref.		
**Challenges associated with physical environment**				
not important, little important, important	13 (32 %)/41	0.29	0.10–0.86	**0.026**
very important	13 (62 %)/21	Ref.		
**Challenges associated with buildings, housing, roads**				
not important, little important, important	17 (40 %)/42	0.31	0.10–1.0	**0.048**
very important	13 (68 %)/19	Ref.		
**Challenges associated with culture**				
not important, little important, important	10 (28 %)/36	0.19	0.06–0.60	**0.004**
very important	16 (67 %)/24	Ref.		

*p*-values ≤ 0.05 are written in bold.

Not being in nature for recreational activities seemed to decrease very good satisfaction with life (Odds Ratio ⁅OR⁆ 0.41, *p* = 0.044, 95 % Confidence interval ⁅95% CI⁆ 0.17–0.98), compared to those who spent time in nature very often. The assessment that challenges of permafrost thawing were not very importantly associated with culture decreased the odds for very good quality of life (OR 0.25, *p* = 0.043, 95 % CI 0.07–1.0) and satisfaction with life (OR 0.19, p = 0.004, 95 % CI 0.06–0.60). Similarly, not acknowledging that challenges of thawing were not very importantly associated with physical environment (OR 0.29, p = 0.026, 95 % CI 0.10–0.86) or housing, buildings and roads (OR 0.31, p = 0.048, 95 % CI 0.10–1.0) seemed to decrease likelihood of very good satisfaction with life (See Table 3).

#### 3.2.3 Open questions

The responses to the open questions were similar to the results of the fieldwork. In addition to the results presented, pollution and the global challenges of climate change were mentioned, and people were aware of the risks related to them. Participants did not know or name any specific needs that could support their ability to face and adapt to changes in their own life due to climate change. Everyday challenges mentioned by the participants were related to personal issues such as finances, challenges regarding individual and family health, family relationships and society, difficulties at work and changes in nature. Some participants noted that the hunting catch from nature were not that good anymore, resulting in financial difficulties and amplifying challenges at work and in nature.

#### 3.2.4 Summary of results

**Figure 2:**
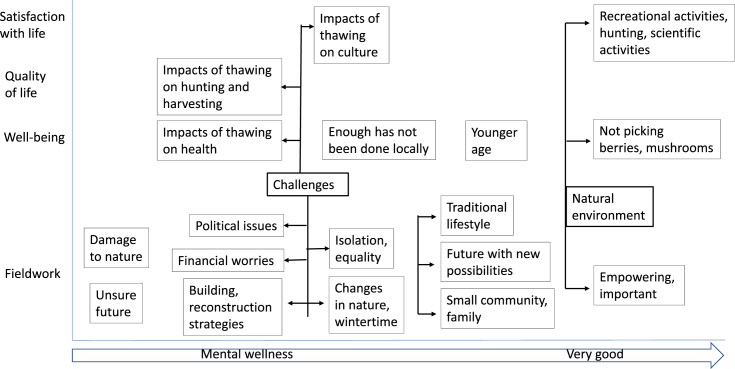
Summary of results.

The main results are presented in Figure 2.

## 4 Discussion

A majority of the participants reported good well-being (58 %), quality of life (71 %) and satisfaction with life (59 %) in the questionnaire survey. The second largest group assessed their well-being (28 %), quality of life (21 %) and satisfaction with life (38 %) as very good. This is supported by the data from the fieldwork – life in a small community was found to be good due to a variety of factors, such as feeling safe. Gender was not significantly associated with well-being, quality of life or satisfaction with life. Altogether 52 males and 48 females answered the questionnaire. More of the questions focused on hunting and harvesting, which may reflect that the research assistant felt it was more natural to interview the men in the community. This occupation has traditionally been - and at the time of the research still was - an occupation for males.

Being of a younger age was associated with very good well-being. Results indicated that in particular participants aged 45–64 years old had lower well-being. This result differs from the findings reported in the current literature in general, that youth in Greenland are suffering from serious mental health problems (Bjerregaard and Larsen, 2015). Based on the results, the local people wished for more Greenlandic health care staff, and speaking solely Greenlandic language at home seemed to decrease very good quality of life or satisfaction with life. The main language spoken in health care is Danish as the majority of the health care workers are from Denmark (Ellsworth and O'Keeffe, 2013), even though the official language is Greenlandic and the second language is Danish (Bjerrregaard and Larsen, 2018). It can be a challenge to receive comprehensive help and support in life in one's own language in Greenland. It may also be challenging to speak to someone in a small community, even to a professional due to issues of access and societal issues, such as gossiping.

Based on the univariate analysis, recognizing that challenges associated with the physical environment or infrastructure are very important seemed to increase satisfaction with life. According to the fieldwork results, these problems were recognized in the participants’ living environment. Still, self-rated well-being, quality of life and satisfaction with life were rated high, and adaptation needs were not significantly associated with the variables of mental wellness. However, based on the results of multivariate analysis, changes in the environment or challenges caused by thawing permafrost were not significantly associated with the dependent variables describing the mental wellness of local people.

Challenges associated with culture were very important, and spending a large amount of time in nature, while engaged in various activities, appear to increase mental wellness. Nature was found to be empowering and traditional ways of life formed an essential part of peoples' lives. These findings are supported by the current literature. Nature has been and still is an important part of Arctic identity. It maintains Arctic peoples’ holistic well-being and needs (Crate et al., 2010). According to Barton and Pretty (2010), nature seems to be one of the key elements supporting well-being and improving self-esteem. Interestingly, picking berries or mushrooms decreased the likelihood of very good well-being. This may suggest that women in this study sample are more likely picking berries and mushrooms in families, and they may face more demands in their roles, resulting in a feeling of burden (Higgins et al., 2010). This, however, did not show up in the results of the fieldwork.

Although the discussion around climate change is growing globally, people responded that climate change as a term in itself was not present in their everyday discussions, and that warming weather was not just seen as a negative impact – rather, it could create new possibilities to support their lives. Similar results have observed by Nuttall (2010), who talks about anticipation in terms of how people are prepared for climate change in Greenland. While climate change brings difficulties it can also create new opportunities, and therefore it is important to understand peoples' anticipation in life (Nuttall 2010). The introduction of new technologies and the modernization of society (e.g new equipment to hunt/fish, travel possibilities, infrastructure), occurring concurrently with climate change, might also, help to counterbalance and facilitate certain aspects of life as they undergo these changes. Another possible explanation is that big and dramatic changes in nature and the environment are not necessarily immediately visible to the community – changes are more remote, even in Greenland. It can also be that permafrost thawing is not a major problem for everyone in the study area, as the communities are located in the zone of continuous/discontinuous permafrost. On the other hand, people have limited or no other option than to adapt to changes over the course of time, and even big changes can be accommodated over time – changes and adaptation are part of Indigenous people's lives. Local people in the study area wanted to maintain a peaceful nature. However, changes in nature and climate were observed with concerns due to risks related to environmental and natural disasters. According to Hjort et al. (2018), permafrost thaw is negatively affecting the environment, especially modern infrastructure, which may become a serious threat in the future.

The results suggested that well-being was reduced when people had the feeling that not enough has been done by local authorities. This finding is similar to the fieldwork observations which showed that people felt they have little power to influence decision-making. This may reflect that global actions and discussions are providing hope but no or limited local policy changes. Generally, numerous challenges related to society and environment will impact the life of Arctic people (Larsen and Fondahl, 2014). According to Waits et al. (2018), the warming climate in the Arctic, combined with more people travelling from different countries, is posing new health risks in the form of infectious diseases. Current COVID-19 pandemic, caused by a zoonotic virus (Contini et al., 2020), has posed serious threats to Arctic people as well. Overall, the warming climate and other societal changes have put pressure on the local population and living habits (Larsen et al., 2015). These elements all have an effect on peoples’ mental health (Cunsolo Willox et al., 2012, 2013a).

The research does have some limitations. First, researchers in the group used several languages: English, Danish and Greenlandic. English was used when it was possible and when interviewees and representatives felt comfortable using it, but an interpreter (Danish speaking) was also used in the meetings where local people spoke Greenlandic. Furthermore, researchers who spoke Danish translated the content into English for other researchers. This process may have limited the possibility to recheck some of the contents that local people expressed, not only due to the use of different languages, but also due to the multidisciplinary research teamwork. Also, notes were shared after the information was filtered through the researcher. This process can impact on the reliability and validity of results. In qualitative research it is known that a researcher's own personal thoughts and experiences can have an effect on how participants' thoughts are understood, and the qualitative analysis process includes a risk of including a researcher's own perspectives (Noble and Smith, 2015). These risks have been acknowledged and researchers have made an effort to limit them when working with the data from fieldwork.

The study sample is rather small, and results cannot be generalized. Research with a larger study sample is required in the future not only due to generalization of results, but also for being able to conduct more powerful and reliable statistical analyses. The statistical analysis aimed to find out which perceived environmental and adaptation factors relate to each dependent variable. Based on the results, adaptation factors are less represented. In addition, answers N/A had a big influence on some of the factors, and therefore created limitations to the statistical analysis. The 95 % confidence intervals of multivariate logistic regression analysis were wide due to the small sample size. Therefore, only the results of the univariate analysis were presented in the results section; however, similar problems occurred in some parts of the models. Despite the small sample size, the results can still offer relevant information for further plans and actions in the field of research and at the policy level. It also shows an example of multidisciplinary research that includes the important voice of communities – a voice that should be involved in research when investigating climate change and its local impacts.

The methods used in this research provide a comprehensive picture of the quality of life and mental wellness of the community participants. Joint co-operation and mutual understanding between researchers and local people could help support both sides to understand issues in a deeper way in order to create a more holistic picture. Discussions of the fieldwork provided a valid structure for researchers to understand the living conditions and life of local people, but it also offered an important opportunity for researchers to inform local people about the project, listen to their thoughts and modify their research plans, if necessary. This kind of approach also helped researchers to work with the questionnaire data and to conduct the statistical analysis. The methods used and teamwork approach of the study supported learning and transdisciplinary cooperation in a deeper way: It provided insight into how we view and understand different issues in science. This certainly requires careful and respectful discussion among researchers in order to understand each other and to find mutually agreed ways and methods to complete joint research. This learning process will continue, together with researchers and community members.

## 5 Conclusions

According to this research, permafrost thaw is not significantly associated with the self-rated well-being, quality of life and satisfaction with life that described the mental wellness of local people. Changes and challenges associated with permafrost thaw are recognized, but they do not negatively affect the lives of local communities in a comprehensive or major way. Traditional lifestyles and occupations are important for Greenlandic people, but new industries will emerge in the future, bringing new opportunities. Nature and the different activities within it are empowering and supporting life. Local Indigenous people have slowly and perhaps even inconspicuously adapted to changes in the environment. It seems that yet uncompleted global actions towards climate change are providing hope. Local people wished to be heard and they have local knowledge that is essential in this field of research. This research provides relevant information about the life and living conditions of people surrounded by climate change which can be useful for further research or at the policy level. More research is needed to achieve a deeper understanding of different aspects of mental wellness, especially in connection to permafrost thaw.

## Declarations

### Author contribution statement

Ulla Timlin: Conceived and designed the experiments; Performed the experiments; Analyzed and interpreted the data; Contributed reagents, materials, analysis tools or data; Wrote the paper.

Jón Haukur Ingimundarson, Leneisja Jungsberg, Joan Nymand Larsen, Johanna Schee, Peter Schweitzer, Arja Rautio: Conceived and designed the experiments; Performed the experiments; Analyzed and interpreted the data; Wrote the paper.

Sofia Kauppila, Tanja Nordström: Analyzed and interpreted the data; Wrote the paper.

### Funding statement

This publication is part of the Nunataryuk project. The project has received funding under the European Union's Horizon 2020 Research and Innovation Program under grant agreement no. 773421.

### Data availability statement

The data that has been used is confidential.

### Declaration of interests statement

The authors declare no conflict of interest.

### Additional information

No additional information is available for this paper.
