# Supplementary material for: Living conditions and mental wellness in a changing climate and environment: focus on community voices and perceived environmental and adaptation factors in Greenland
Source: Heliyon. 2021 Apr 30;7(4):e06862. doi: 10.1016/j.heliyon.2021.e06862 (PMC8105633; doi:10.1016/j.heliyon.2021.e06862)
Supplement: Table A.1 [file mmc1.docx]

**Table 1.** (Supplement) Variables describing mental wellness and associations with background variables (*p* = <0.1, n = 100)

|  | **Well-being** | | | | | | | | | | | | |
| --- | --- | --- | --- | --- | --- | --- | --- | --- | --- | --- | --- | --- | --- |
|  | **bad, OK** | | **good** | | | **very good** | |  | **not very good** | | **very good** | |  |
|  | n | (%) | n | (%) | | n | (%) | ***p*-value** | n | (%) | n | (%) | ***p*-value** |
| **Age** |  |  |  |  | |  |  |  |  |  |  |  |  |
| 18-24 | 3 | (21) | 5 | (9) | | 2 | (7) | **0.043** | 8 | (11) | 2 | (7) | **0.031** |
| 25-34 | 0 | (0) | 9 | (15) | | 11 | (39) |  | 9 | (12,5) | 11 | (39) |  |
| 35-44 | 3 | (21) | 6 | (10) | | 2 | (7) |  | 9 | (12,5) | 2 | (7) |  |
| 45-54 | 3 | (21) | 17 | (29) | | 8 | (29) |  | 20 | (28) | 8 | (29) |  |
| 55-64 | 4 | (29) | 16 | (28) | | 2 | (7) |  | 20 | (28) | 2 | (7) |  |
| ≥ 65 | 1 | (8) | 5 | (9) | | 3 | (11) |  | 6 | (8) | 3 | (11) |  |
|  | **Quality of life** | | | | | | | | | | | |  |
|  | **bad, OK** | | **good** | | **very good** | | |  | **not very good** | | **very good** | |  |
|  | n | (%) | n | (%) | | n | (%) | ***p*-value** | n | (%) | n | (%) | ***p*-value** |
| **Language at home** |  |  |  |  | |  |  |  |  |  |  |  |  |
| Greenlandic | 7 | (88) | 68 | (96) | | 15 | (71) | **0.004** | 75 | (95) | 15 | (71) | **0.001** |
| Danish | 0 | (0) | 0 | 0 | | 5 | (24) |  | 4 | (5) | 5 | (24) |  |
| Both | 1 | (12 | 3 | (4) | | 1 | (5) |  | 0 | (0) | 1 | (5) |  |
| **Main profession** |  |  |  |  | |  |  |  |  |  |  |  |  |
| Public sector | 2 | (25) | 25 | (35) | | 11 | (52) | 0.065 | 27 | (34) | 11 | (52) | **0.030** |
| Private sector | 1 | (2) | 21 | (30) | | 9 | (43) |  | 22 | (28) | 9 | (43) |  |
| Hunter / fisherman | 2 | (25) | 8 | (11) | | 1 | (5) |  | 10 | (12) | 1 | (5) |  |
| Not employed | 3 | (38) | 17 | (24) | | 0 | (0) |  | 20 | (26) | 0 | (0) |  |
|  | **Satisfaction with life** | | | | | | | | | | | |  |
|  | **bad, OK** | | **good** | | | **very good** | |  | **not very good** | | **very good** | |  |
|  | n | (%) | n | (%) | | n | (%) | ***p*-value** | n | (%) | n | (%) | ***p*-value** |
| **Language at home** |  |  |  |  | |  |  |  |  |  |  |  |  |
| Greenlandic | 3 | (100) | 57 | (97) | | 30 | (79) | **0.031** | 60 | (97) | 30 | (79) | **0.004** |
| Danish | 0 | 0 | 0 | 0 | | 5 | (13) |  | 0 | (0) | 5 | (13) |  |
| Both | 0 | 0 | 2 | (3) | | 3 | (8) |  | 2 | (2) | 3 | (8) |  |
| **Main profession** |  |  |  |  | |  |  |  |  |  |  |  |  |
| Public sector | 1 | (33,3) | 2 | (36) | | 16 | (42) | **0.031** | 22 | (35) | 16 | (42) | **0.004** |
| Private sector | 1 | (33,3) | 13 | (22) | | 17 | (45) |  | 14 | (23) | 17 | (45) |  |
| Hunter / fisherman | 0 | 0 | 7 | (12) | | 4 | (11) |  | 7 | (11) | 4 | (11) |  |
| Not employed | 1 | (33,3) | 18 | (30) | | 1 | (2) |  | 19 | (31) | 1 | (2) |  |
