# Supplementary material for: Living conditions and mental wellness in a changing climate and environment: focus on community voices and perceived environmental and adaptation factors in Greenland
Source: Heliyon. 2021 Apr 30;7(4):e06862. doi: 10.1016/j.heliyon.2021.e06862 (PMC8105633; doi:10.1016/j.heliyon.2021.e06862)
Supplement: Table A.2 [file mmc2.docx]

**Table 2.** (Supplement) Variables describing mental wellness and associations with environmental variables (*p* = ≤0.1, n = 100)

|  | **Well-being** | | | | | | | | | | | | | | | |
| --- | --- | --- | --- | --- | --- | --- | --- | --- | --- | --- | --- | --- | --- | --- | --- | --- |
|  | **bad, OK** | | | **good** | | | **very good** | |  | **not very good** | | | **very good** | | |  |
|  | n | (%) | | n | | (%) | n | (%) | ***p*-value** | n | (%) | n | | (%) | | ***p*-value** |
| **Being in nature for picking berries, mushrooms**  (n = 99) |  |  | |  | |  |  |  |  |  |  |  | |  | |  |
| never, rarely | 2 | (14) | | 13 | | (23) | 12 | (43) | 0.081 | 15 | (21) | 15 | | (43) | | **0.011** |
| sometimes, very often | 12 | (86) | | 44 | | (77) | 15 | (54) |  | 31 | (44) | 15 | | (54) | |  |
| N/A | 0 | 0 | | 0 | | 0 | 1 | (3) |  |  |  |  | |  | |  |
| never, rarely, sometimes | 5 | (36) | | 26 | | (46) | 19 | (68) | 0.069 | 31 | (44) | 19 | | (68) | | **0.012** |
| very often | 9 | (64) | | 31 | | (54) | 8 | (29) |  | 40 | (56) | 8 | | (29) | |  |
| N/A | 0 | 0 | | 0 | | 0 | 1 | (3) |  | 0 | (0) | 1 | | (3) | |  |
| **Challenges are associated with health** (n = 99) |  |  | |  | |  |  |  |  |  |  |  | |  | |  |
| not important, little important | 0 | 0 | | 4 | | (7) | 7 | (25) | 0.055 | 4 | (6) | 7 | | (25) | | **0.021** |
| important, very important | 7 | (54) | | 22 | | (38) | 8 | (29) |  | 29 | (41) | 8 | | (29) | |  |
| N/A | 6 | (46) | | 23 | | (55) | 13 | (46) |  | 38 | (53) | 13 | | (46) | |  |
| **Challenges are associated with buildings, roads, housing** (n = 97) |  |  | |  | |  |  |  |  |  |  |  | |  | |  |
| not important, little important |  |  | |  | |  |  |  |  | 4 | (6) | 5 | | (18) | | 0.086 |
| important, very important |  |  | |  | |  |  |  |  | 37 | (53) | 15 | | (56) | |  |
| N/A |  |  | |  | |  |  |  |  | 29 | (41) | 7 | | (26) | |  |
|  | **Quality of life** | | | | | | | | | | | | |  | | |
|  | **bad, OK** | | | **good** | | | **very good** | |  | **not very good** | | | **very good** | | |  |
|  | n | (%) | | n | | (%) | n | (%) | ***p*-value** | n | (%) | n | | (%) | | ***p*-value** |
| **Being in nature for economic activities** (n = 99) |  |  | |  | |  |  |  | **0.034** |  |  |  | |  | |  |
| never, rarely, sometimes | 5 | (62) | | 66 | | (93) | 17 | (81) |  |  |  |  | |  | |  |
| very often | 2 | (25) | | 4 | | (6) | 4 | (19) |  |  |  |  | |  | |  |
| N/A | 1 | (13) | | 1 | | (1) | 0 | 0 |  |  |  |  | |  | |  |
| **Challenges are associated with culture** (n = 99) |  |  | |  | |  |  |  |  |  |  |  | |  | |  |
| not important, little important, important | 0 | 0 | | 32 | | (45) | 4 | (20) | **0.019** |  |  |  | |  | |  |
| very important | 2 | (25) | | 14 | | (29) | 8 | (40) |  |  |  |  | |  | |  |
| N/A | 6 | (75) | | 25 | | (35) | 8 | (40) |  |  |  |  | |  | |  |
| **Challenges are associated with buildings, roads, housing** (n = 97) |  |  | |  | |  |  |  |  |  |  |  | |  | |  |
| not important, little important, important |  |  | |  | |  |  |  |  | 34 | (44) | 8 | | (42) | | 0.066 |
| very important |  |  | |  | |  |  |  |  | 12 | (15) | 7 | | (37) | |  |
| N/A |  |  | |  | |  |  |  |  | 32 | (41) | 4 | | (21) | |  |
|  | **Satisfaction with life** | | | | | | | | | | | | | |  | |
|  | **bad, OK** | | | **good** | | | **very good** | |  | **not very good** | | | **very good** | | |  |
|  | n | (%) | | n | | (%) | n | (%) | ***p*-value** | n | (%) | n | | (%) | | ***p*-value** |
| **Being in nature for hunting** (n = 100) |  |  | |  | |  |  |  |  |  |  |  | |  | |  |
| never, rarely |  |  | |  | |  |  |  |  | 35 | (57) | 14 | | (37) | | 0.061 |
| sometimes, very often |  |  | |  | |  |  |  |  | 27 | (43) | 23 | | (60) | |  |
| N/A |  |  | |  | |  |  |  |  | 0 | 0 | 1 | | (3) | |  |
| **Being in nature for recreation activities** (n = 100) |  |  | |  | |  |  |  |  |  |  |  | |  | |  |
| never, rarely, sometimes |  |  | |  | |  |  |  |  | 29 | (47) | 10 | | (26) | | 0.057 |
| very often |  |  | |  | |  |  |  |  | 33 | (53) | 28 | | (74) | |  |
| **Being in nature for scientific activities** (n = 97) |  |  | |  | |  |  |  |  |  |  |  | |  | |  |
| never, rarely | 2 | (67) | | 54 | | (96) | 33 | (87) | 0.072 |  |  |  | |  | |  |
| sometimes, very often | 21 | (39) | | 0 | | 0 | 5 | (13) |  |  |  |  | |  | |  |
| N/A | 0 | 0 | | 2 | | (4) | 0 | 0 |  |  |  |  | |  | |  |
| never, rarely |  |  | |  | |  |  |  |  | 56 | (95) | 33 | | (87) | | **0.027** |
| sometimes, very often |  |  | |  | |  |  |  |  | 1 | (2) | 5 | | (13) | |  |
| N/A |  |  | |  | |  |  |  |  | 2 | (3) | 0 | | (0) | |  |
| **Challenges are associated with culture** (n = 99) |  |  | |  | |  |  |  |  |  |  |  | |  | |  |
| not important, little important, important |  |  | |  | |  |  |  |  | 26 | (43) | 10 | | (26) | | **0.005** |
| very important |  |  | |  | |  |  |  |  | 8 | (13) | 16 | | (42) | |  |
| N/A |  |  | |  | |  |  |  |  | 27 | (44) | 12 | | (32) | |  |
|  |  |  | |  | |  |  |  |  |  |  |  | |  | |  |
|  |  |  | |  | |  |  |  |  |  |  |  | |  | |  |
| **Table 2. Continue** |  |  | |  | |  |  |  |  |  |  |  | |  | |  |
|  | **bad, OK** | | | | **good** | | **very good** | | ***p*-value** | **not very good** | | | **very good** | | | ***p*-value** |
| **Challenges are associated with housing, buildings, roads** (n=97) |  | |  | |  |  |  |  |  |  |  |  | |  | |  |
| not important, little important | 1 | | (33) | | 5 | (9) | 3 | (8) | **0.014** | 6 | (10) | 3 | | (8) | | **0.007** |
| important, very important | 2 | | (67) | | 23 | (40) | 27 | (73) |  | 25 | (42) | 27 | | (73) | |  |
| N/A | 0 | | 0 | | 29 | (51) | 7 | (19) |  | 29 | (48) | 7 | | (19) | |  |
| not important, little important, important | 2 | | (67) | | 23 | (40) | 17 | (46) | **0.002** | 25 | (42) | 17 | | (46) | | **0.002** |
| very important | 1 | | (33) | | 5 | (9) | 13 | (35) |  | 6 | (10) | 13 | | (35) | |  |
| N/A | 0 | | 0 | | 29 | (51) | 7 | (19) |  | 29 | (48) | 7 | | (19) | |  |
| **Challenges are associated with physical environment** (n=99) |  | |  | |  |  |  |  |  |  |  |  | |  | |  |
| not important, little important, important |  | |  | |  |  |  |  |  | 28 | (46) | 13 | | (34) | | **0.049** |
| very important |  | |  | |  |  |  |  |  | 8 | (13) | 13 | | (34) | |  |
| N/A |  | |  | |  |  |  |  |  | 25 | (41) | 12 | | (32) | |  |
| **Challenges are associated with economic activities** (n=99) |  | |  | |  |  |  |  |  |  |  |  | |  | |  |
| not important, little important | 2 | | (67) | | 2 | (3) | 1 | (3) | **0.001** |  |  |  | |  | |  |
| important, very important | 1 | | (33) | | 24 | (42) | 23 | (60) |  |  |  |  | |  | |  |
| N/A | 0 | | 0 | | 32 | (55) | 14 | (37) |  |  |  |  | |  | |  |
| not important, little important, important | 3 | | (100) | | 20 | (35) | 15 | (40) | **0.043** |  |  |  | |  | |  |
| very important | 0 | | 0 | | 6 | (10) | 9 | (23) |  |  |  |  | |  | |  |
| N/A | 0 | | 0 | | 32 | (55) | 14 | (37) |  |  |  |  | |  | |  |
