# Supplementary material for: Living conditions and mental wellness in a changing climate and environment: focus on community voices and perceived environmental and adaptation factors in Greenland
Source: Heliyon. 2021 Apr 30;7(4):e06862. doi: 10.1016/j.heliyon.2021.e06862 (PMC8105633; doi:10.1016/j.heliyon.2021.e06862)
Supplement: Table A.3 [file mmc3.docx]

**Table 3.** (Supplement) Associations to variables describing mental wellness and thawing permafrost and adaptation (p = ≤0.1)

|  | **Wellbeing** | | | | | | | | | | | | |
| --- | --- | --- | --- | --- | --- | --- | --- | --- | --- | --- | --- | --- | --- |
|  | **bad, OK** | | **good** | | **very good** | |  | **not very good** | | **very good** | | |  |
|  | n | (%) | n | (%) | n | (%) | ***p*-value** | n | (%) | n | (%) | | ***p*-value** |
| **Thawing is connected to problems** (n = 22) |  |  |  |  |  |  |  |  |  |  |  | |  |
| financial | 0 | 0 | 7 | (64) | 1 | (12,5) | 0.061 |  |  |  |  | |  |
| spiritual | 1 | (33,3) | 0 | 0 | 0 | 0 |  |  |  |  |  | |  |
| safety | 0 | 0 | 2 | (18) | 2 | (25) |  |  |  |  |  | |  |
| political | 1 | (33,3) | 0 | 0 | 2 | (25) |  |  |  |  |  | |  |
| health | 1 | (33,3) | 2 | (18) | 2 | (25) |  |  |  |  |  | |  |
| other | 0 | 0 | 0 | 0 | 1 | (12,5) |  |  |  |  |  | |  |
| **Which ways community is best prepared to adapt permafrost thawing** (n = 82) |  |  |  |  |  |  |  |  |  |  |  | |  |
| hunting, fishing activities | 2 | (25) | 8 | (16) | 3 | (13) | **0.003** | 10 | (17) | 3 | (13) | | **0.005** |
| housing | 0 | 0 | 7 | (14) | 1 | (4) |  | 7 | (12) | 1 | (4) | |  |
| health | 0 | 0 | 4 | (8) | 8 | (35) |  | 4 | (7) | 8 | (35) | |  |
| traditional knowledge | 2 | (25) | 0 | 0 | 3 | (13) |  | 2 | (3) | 3 | (13) | |  |
| I don’t’ know | 4 | (50) | 32 | (63) | 8 | (35) |  | 36 | (61) | 8 | (35) | |  |
| **Enough done to adapt / face the impacts, by individuals** (n = 86) |  |  |  |  |  |  |  |  |  |  |  | |  |
| no |  |  |  |  |  |  |  | 46 | (75) | 16 | (64) | | 0.062 |
| somewhat |  |  |  |  |  |  |  | 10 | (16) | 9 | (36) | |  |
| yes |  |  |  |  |  |  |  | 5 | (8) | 0 | 0 | |  |
| **Enough done to adapt / face the impacts, by local authorities** (n = 85) |  |  |  |  |  |  |  |  |  |  |  | |  |
| no | 10 | (83) | 40 | (83) | 17 | (68) | 0.087 | 50 | (84) | 17 | (68) | | 0.057 |
| somewhat | 0 | 0 | 5 | (10) | 7 | (28) |  | 5 | (8) | 7 | (28) | |  |
| yes | 2 | (17) | 3 | (6) | 1 | (4) |  | 5 | (8) | 1 | (4) | |  |
|  | **Quality of life** | | | | | | | | | |  | | |
|  | **bad, OK** | | **good** | | **very good** | |  | **not very good** | | **very good** | | |  |
|  | n | (%) | n | (%) | n | (%) | ***p*-value** | n | (%) | n | (%) | | ***p*-value** |
| **Main cause of the thawing ground** (n = 5) |  |  |  |  |  |  |  |  |  |  |  | |  |
| option chosen: God | 3 | (38) | 2 | (3) | 0 | 0 | **0.003** |  |  |  |  | |  |
| option was not chosen | 5 | (62) | 69 | (97) | 21 | (100) |  |  |  |  |  | |  |
| **Lost picking spots because of flooded or submerged ground** (n = 99) |  |  |  |  |  |  |  |  |  |  |  | |  |
| yes | 3 | (38) | 3 | (4) | 0 | 0 | **0.004** |  |  |  |  | |  |
| no | 5 | (62) | 67 | (96) | 21 | (100) |  |  |  |  |  | |  |
| **Thawing causes problems** (n = 100) |  |  |  |  |  |  |  |  |  |  |  | |  |
| yes | 3 | (37,5) | 14 | (20) | 4 | (19) | **0.034** |  |  |  |  | |  |
| no | 2 | (25) | 47 | (66) | 18 | (81) |  |  |  |  |  | |  |
| N/A | 3 | (37,5) | 10 | (14) | 0 | 0 |  |  |  |  |  | |  |
|  | **Satisfaction with life** | | | | | | | | | | |  | |
|  | **bad, OK** | | **good** | | **very good** | |  | **not very good** | | **very good** | |  | |
|  | n | (%) | n | (%) | n | (%) | ***p*-value** | n | (%) | n | (%) | | ***p*-value** |
| **Main cause of the thawing ground** (n = 13) |  |  |  |  |  |  |  |  |  |  |  | |  |
| option chosen: Decrease in snow depth | 2 | (67) | 5 | (9) | 6 | (16) | **0.020** |  |  |  |  | |  |
| option was not chosen | 1 | (33) | 54 | (91) | 32 | (84) |  |  |  |  |  | |  |
| **Main cause of the thawing ground** (n = 5) |  |  |  |  |  |  |  |  |  |  |  | |  |
| option chosen: God | 1 | (33) | 4 | (7) | 0 | 0 | **0.034** |  |  |  |  | |  |
| option was not chosen | 2 | (67) | 55 | (93) | 38 | (100) |  |  |  |  |  | |  |
