# Supplementary material for: Living conditions and mental wellness in a changing climate and environment: focus on community voices and perceived environmental and adaptation factors in Greenland
Source: Heliyon. 2021 Apr 30;7(4):e06862. doi: 10.1016/j.heliyon.2021.e06862 (PMC8105633; doi:10.1016/j.heliyon.2021.e06862)
Supplement: Table A.4 [file mmc4.docx]

**Table 4. Supplement.**  Multivariate analysis: Associations between variables describing mental wellness and perceived environmental factors (*p* = <0.06)

|  | **yes (n / %) / total** | **OR** | **95 % CI** | ***p-*value** |
| --- | --- | --- | --- | --- |
| **Variables** | **Very good well-being** |  |  |  |
| **Being in nature for picking berries, mushrooms^a^** |  |  |  |  |
| never, rarely sometimes | 19 (38 %) / 50 | 3.18 | 1.13-8.94 | **0.028** |
| very often | 8 (17 %) / 48 | Ref. |  |  |
| ***Model statistics*** *-AIC 112.227; R^2^ 0.181;*  *HL:* χ^2^ *7.747, df 8, p 0.46* | | | | |
| **Challenges associated with health^a^** |  |  |  |  |
| not important, little important | 7 (64 %) / 11 | 5.43 | 1.07 – 27.55 | **0.041** |
| important, very important | 8 (22 %) / 37 | Ref. |  |  |
| ***Model statistics*** *AIC 56.045; R^2^ 0.346:*  *HL:* χ^2^ *10.822, df 8, p 0.21* | | | | |
|  | **Very good quality of life** |  |  |  |
| **Challenges associated with**  **hunting and harvesting^b^** |  |  |  |  |
| not important, little important, important | 11 (26 %) / 43 | 20.08 | 1.63 – 247.70 | **0.019** |
| very important | 1 (6 %) / 18 | Ref. |  |  |
| ***Model statistics*** *AIC 53.558; R^2^ 0.345;*  *HL:* χ^2^ *3.328, df 6, p 0.77* | | | | |
|  | **Very good satisfaction with life** |  |  |  |
| **Being in nature for recreation activities^a^** |  |  |  |  |
| never, rarely, sometimes | 10 (26 %) / 39 | 0.36 | 0.13-1.01 | 0.052 |
| very often | 28 (46 %) / 61 | Ref. |  |  |
| ***Model statistics*** *AIC 112.156; R^2^ 0.359;*  *HL:* χ^2^ *4.050, df 8, p 0.85* | | | | |
| **Being in nature for scientific activities^a^** |  |  |  |  |
| never, rarely | 33 (37 %) / 89 | 0.06 | 0.003-1.10 | 0.058 |
| sometimes, very often | 5 (83 %) / 6 | Ref. |  |  |
| ***Model statistics*** *AIC 105.428; R^2^ 0.391;*  *HL:* χ^2^ *3.854, df 6, p 0.70* | | | | |
| **Challenges associated with culture^a^** |  |  |  |  |
| not important, little important, important | 10 (28 %) / 36 | 0.19 | 0.05 – 0.67 | **0.010** |
| very important | 16 (67 %) / 24 | Ref. |  |  |
| ***Model statistics*** *AIC 72.214; R^2^ 0.379;*  *HL:* χ^2^ *8.074, df 8, p 0.43* | | | | |
| **^a^** Adjusted for age, gender, language, employment situation  **^b^** Adjusted for age, gender, language  **^c^** Adjusted for age, language, employment situation  Abbreviations: AIC: Akaike information criterion; R^2^: Nagelkerke R^2^ ; Hosmer-Lemeshow goodness-of-fit test (HL) | | | | |
